# Yixintai treats chronic heart failure in rats by regulating gut microbiota and bile acid

**DOI:** 10.3389/fmicb.2025.1672313

**Published:** 2025-11-26

**Authors:** Min Shi, Hui Yuan, Chengxin Liu, Jiaming Wei, Ziyan Wang, Aisi Huang, Qinghua Zeng, Ya Li, Zhihua Guo

**Affiliations:** 1 Hunan University of Chinese Medicine, Changsha, China; 2 Provincial Key Laboratory of TCM Diagnostics, Hunan University of Chinese Medicine, Changsha, China; 3 First Clinical College of Chinese Medicine, Hunan University of Chinese Medicine, Changsha, China; 4 The Second Clinical College of Chinese Medicine, The Second Affiliated Hospital of Hunan University of Chinese Medicine, Changsha, China; 5 School of Pharmacy, Hunan University of Chinese Medicine, Changsha, China

**Keywords:** chronic heart failure, Yixintai, gut microbiota, bile acid, TGR5

## Abstract

**Introduction:**

Yixintai (YXT) medicine for chronic heart failure (CHF), has demonstrated safety and efficacy in the treatment of CHF. However, its precise mechanistic actions require further elucidation.

**Methods:**

This study identified components in YXT using the UHPLC-QE-MS technique. A rat CHF model was created by ligating the left anterior descending coronary artery and an inflammatory injury model was induced in H9c2 cells using lipopolysaccharide (LPS) to evaluate the efficacy of YXT. After YXT treatment, changes in fecal gut microbiota and serum BAs profiles in rats were evaluated utilizing 16S rRNA sequencing and UHPLC-MS/MS techniques. Additionally, western blot (WB) and polymerase chain reaction (PCR) assays were conducted to assess the expression levels of TGR5 in both myocardial tissue and H9c2 cells. Cyclic adenosine monophosphate (cAMP), B-type natriuretic peptide (BNP), interleukin-1 beta (IL-1β), interleukin-6 (IL-6), and tumor necrosis factor-alpha (TNF-α) levels were also measured using enzyme-linked immunosorbent assay (ELISA).

**Results:**

In total, 1049 components were identified in YXT. YXT treatment effectively attenuated the inflammatory reaction, reduced serum BNP levels, alleviated the pathological changes in the colon and myocardium, and improved cardiac function in CHF rats. YXT treatment significantly improved gut microbiota diversity in CHF rats, enhancing beneficial bacterial populations and serum bile acid levels, while reducing the abundance of detrimental bacteria. Furthermore, YXT treatment enhanced TGR5 expression in the myocardial tissue and H9c2 cells of CHF rats.

**Discussion:**

These findings suggest that YXT exerts its therapeutic benefits by reshaping the gut microbiota, modulating bile acid metabolism, and activating TGR5.

## 1 Introduction

Chronic heart failure (CHF) is a long-term condition that can stabilize, worsen, or lead to decompensation. As a severe outcome of various cardiovascular disorders, CHF manifests through dyspnea, fatigue, and fluid retention. The substantial disease burden of CHF, driven by its elevated rates of morbidity and mortality, underscores its global healthcare challenge (Baman and Ahmad, 2020). Afflicting more than 60 million individuals worldwide, heart failure (HF) represents a pervasive clinical entity that severely compromises daily functioning and well-being. Demographic aging and increasing chronic illnesses have contributed to escalating HF incidence (Savarese et al., 2023).

Heart failure poses a considerable risk to human health, often leading to poor prognoses and elevated mortality rates. Over 56 million people globally have HF, with a five-year survival rate below 50% post-diagnosis. As populations age and new treatments prolong life, HF cases are expected to increase by 46% by 2030, heavily straining healthcare systems. Despite a decline in overall HF mortality over the past decade, the five-year mortality rate is still a concerning 75% (Khan et al., 2024). While modern medicine has made notable strides in the prevention and management of CHF, prolonged use of pharmacological therapies can lead to various challenges, including adverse drug reactions and reduced quality of life. Furthermore, the lack of personalized treatment strategies and the relatively simplistic targeting of conventional therapies have emerged as notable limitations (Wohlfahrt et al., 2023; Bonfioli et al., 2025). Traditional Chinese Medicine (TCM)’s multi-target, multi-pathway approach has shown efficacy in improving symptomatic relief, functional capacity, and life quality in CHF (Wang et al., 2017). YXT, a traditional Chinese herbal formulation, has shown promising clinical efficacy in managing CHF, with its safety and therapeutic potential thoroughly validated through extensive research (Wang et al., 2024). YXT comprises herbal ingredients such as *Astragalus membranaceus*, *Salvia miltiorrhiza*, and *Carthamus tinctorius*, which are rich in bioactive compounds with therapeutic potential properties CHF. Genistein and formononetin are the primary effective components in YXT for treating HF. Research indicates that these compounds can markedly alleviate HF symptoms post-myocardial infarction and enhance cardiac function and ultrastructural integrity (Sangeethadevi et al., 2022; Wang M. N. et al., 2022; Wang X. et al., 2022).

The gut microbiota is integral to digestion and nutrient assimilation, providing essential energy to the host. Moreover, it functions as an endocrine organ, generating bioactive compounds and metabolites, including bile acid (BA), short-chain fatty acids (SCFAs), and trimethylamine oxide (TMAO). These metabolites are crucial for metabolic processes and influence numerous physiological and pathological pathways (Tang et al., 2019; Yu et al., 2023). According to the ‘gut hypothesis,’ the gut microbiota is vital in managing CHF conditions (Nagatomo and Tang, 2015). In CHF, conditions such as ischemia, hypoxia, and edema can impair the intestinal lining, resulting in increased permeability and dysbiosis. This imbalance facilitates bacterial translocation, endotoxemia, and the cascade of pro-inflammatory mediators, which triggers systemic inflammation. Such inflammation adversely affects vascular endothelial function, impedes blood flow, disrupts nutrient supply, and contributes to multi-organ dysfunction, worsening CHF progression. Furthermore, the dynamic interplay between the host and gut microbiota is essential for BA metabolism and signaling, which are crucial for sustaining metabolic health (Nie et al., 2015; Chen et al., 2020). A recent study exploring the link between BA and cardiac function suggests that these compounds are significantly involved in CHF (Mayerhofer et al., 2017). Evidence has also shown that serum BA levels in CHF patients markedly differ from those in healthy individuals, suggesting that alterations in the gut microbiota-BA axis could play a role in the development of CHF (Winston and Theriot, 2020). BA regulate cardiovascular function by serving as signaling molecules, interacting with G protein-coupled receptors, including Takeda G protein-coupled receptor 5 (TGR5), and nuclear receptors such as the farnesoid X receptor (FXR) and pregnane X receptor/steroid and xenobiotic receptor (PXR/SXR) (Tang et al., 2019). Activation of cell surface receptor TGR5 by BA has been demonstrated to trigger protective responses in cardiac cells. In murine models, such effects enhance myocardial sensitivity to stimuli, boost contractility, and promote hemodynamic adaptation (Luqman et al., 2024).

To established a CHF model, rats underwent left anterior descending coronary artery ligation combined with a restricted diet. An inflammatory injury model was also induced in H9c2 cells using lipopolysaccharide (LPS). We investigated YXT’s cardioprotective mechanisms, focusing on its effects on gut microbiota and related BA metabolites in CHF rats.

## 2 Materials and methods

### 2.1 Drug preparation and analysis

YixinTai (YXT) comprises eight TCM herbs, sourced from EFONG Pharmaceutical Ltd., in (Guangdong, China). The herbs included in this formulation are Huangqi (*Astragalus membranaceus* (Fisch.) Bunge, Cat. No. 1051693), Danshen (*Salvia miltiorrhiza Bunge*, Cat. No. 1080473), Honghua (*Carthamus tinctorius L*, Cat. No. 1081223), Renshen (*Panax ginseng C*. A. Mey, Cat. No. 1042233), Zexie (*Alisma plantago-aquatica L*, Cat. No. 1081173), Fuling (*Poria cocos* (Schw) Wol, Cat. No. 1071523), Zhuling (*Polyporus umbellatus* (Pers.) Fr, Cat. No. 1051953), and Tinglizi (Draba nemorosa L. (Cat. No. 1042123).

Sprague-Dawley (SD) rats (*n* = 10) were randomized into control or YXT-treated (5.6 g/kg/day) groups. After 10 days, blood was collected via abdominal aorta under pentobarbital anesthesia. Serum was isolated, heat-inactivated (56 °C, 30 min), and stored at −80°C. YXT samples were homogenized in boiling water, vortexed, and centrifuged. Supernatant (300 μL) was mixed with methanol:water (4:1, containing IS), vortexed, sonicated (ice-bath), then stored at −40°C (1 h). After centrifugation and filtration (0.22 μm), QC aliquots were stored at −80°C. Samples were analyzed using UPLC (BEH C18 column; 0.1% formic acid mobile phase; 5 μL injection) with gradient elution (85%→25% A in 11 min). MS (Q Exactive) operated in FullScan-ddMS2 mode (70,000/17,500 resolution; ± 4.0/−3.6 kV spray voltage). Data were processed via XCMS (peak alignment, feature extraction) and annotated using MS/MS matching against a custom database.

### 2.2 Establishment and group treatment of a CHF rat model

Sixty SD rats were randomly allocated into two groups: the sham surgery group (*n* = 10) and the ligation group (*n* = 50). Anesthesia was induced with 2% sodium pentobarbital at a dose of 0.28 mL per 100 g body weight (Merck KGaA, Cat. No. P3761) to facilitate left anterior descending coronary artery ligation (Samsamshariat et al., 2005). The Sham group underwent the same surgical procedure without ligation. Post-surgery electrocardiograms confirmed successful ligation with elevated ST-T segments in the thoracic lead. Rats with successful ligation were placed on dietary restriction to establish the CHF model. After 4 weeks, cardiac ultrasound measurements revealed a left ventricular ejection fraction (EF) of 50%, confirming the successful induction of the CHF model. The rats with confirmed CHF were then randomly allocated to six groups: sham group (Sham), model group (Model), low-dose YXT granule group (Low, 1.4 g/kg), medium-dose YXT granule group (Middle, 2.8 g/kg), high-dose YXT granule group (High, 5.6 g/kg), and trimetazidine group (TMZ, 0.01 g/kg). The Model group received an equivalent volume of distilled water, administered once daily. After 4 weeks of therapeutic intervention, rats were humanely sacrificed under anesthesia, allowing complete exposure of their thoracic and abdominal cavities. Various biological specimens, including serum, colonic tissue, myocardial tissue, and fecal content from the colon, were collected for analysis.

### 2.3 Cell culture and group treatment

A rat cardiomyocyte cell line, H9c2, obtained from Wuhan Procell Life Technology Co., Ltd., (Wuhan, China), was cultured in high-glucose Dulbecco’s Modified Eagle Medium (DMEM, Cat No. PM150210, Procell) supplemented with 10% fetal bovine serum (FBS, Cat No. 164210-50, Procell) and 1% (v/v) penicillin/streptomycin (Cat No. PB180120, Procell) at 37°C in a 5% CO2 incubator. Twenty healthy adult male rats were randomly divided into two groups: the YXT group (5.6 g/kg/day) and the control group (equivalent saline), with gavage administration lasting 10 days. Two hours post-final gavage, rat serum was collected, and the supernatant was centrifuged and filtered through a 0.22 μm microwell filter membrane. The serum was then refrigerated at 56°C for 30 min, followed by reheating in a 37 °C constant temperature bath before experimentation. H9c2 cells were cultured until reaching 70–80% confluence. Subsequently, the cells were randomly divided into the nine groups: CON group (10% FBS), LPS group (1 μg/L; Cat No. L2630-10MG, Sigma), YXT drug serum group (10%), Cholic acid (CA) group (100 μg/mL; Cat. No. HY-N0324, MCE), Deoxycholic Acid (DCA) group (150 μg/mL; Cat. No. HY-N0593, MCE), Chenodeoxycholic Acid (CDCA) group (150 μg/mL; Cat. No. HY-76847, MCE), Lithocholic Acid (LCA) group (30 μg/mL; Cat. No. HY-B0172, MCE), TGR5 inhibitor group (100 μg/mL SBI-115; Cat. No. HY-111534, MCE), and TGR5 agonist group (100 μg/mL INT-77; Cat. No. HY-15677, MCE). All groups, except for the CON group, were treated with 1 μg/L LPS for 12 h. Afterward, each group was treated with respective drug concentrations for 24 h. Following treatment, cell pellets and supernatants were collected for further analysis.

### 2.4 Indicator detection and methods

#### 2.4.1 Echocardiographic assessment of cardiac function

After a four-week treatment, rats were anesthetized with isoflurane (Cat. No. 22090401, Shenzhen Rayward Life Technology Co., Ltd., Shenzhen, China). Subsequently, left ventricular EF and FS were examined utilizing a high-resolution small animal echocardiography system (Feieno Technology Co., Ltd., Version: 6 LAB) to assess the subjects’ cardiac function.

#### 2.4.2 Histopathological analysis

After fixation in 10% paraformaldehyde, colon and cardiac tissues underwent dehydration followed by paraffin embedding. Sections were stained with hematoxylin and eosin (HE) and histological changes in colon and myocardial tissues were assessed under an optical microscope (Eclipse E100, Nikon, Japan).

#### 2.4.3 16 S rRNA gene sequencing

Bacterial genomic DNA was extracted from rat fecal samples using the cetyltrimethylammonium bromide (CTAB) method. The target sequence was amplified with 16S rRNA gene-specific primers (5′-AGRGTTTGATYNTGGCTCAG-3′ and 5′-TASGGHTACCTTGTTASGACTT-3′). The PCR product was purified using AMPure XT beads (Beckman Coulter Genomics, Danvers, MA, USA) and quantitatively assessed with Qubit (Invitrogen, USA). The purified PCR products were sequenced using an Agilent 2100 Bioanalyzer library quantification kit (Agilent, USA) and Illumina (KapaBiosciences, Woburn, MA, USA) to acquire the 16S rRNA gene sequences. Subsequently, the data underwent splicing and filtering for species diversity analysis and sequence alignment.

#### 2.4.4 BA profiling

A 100 μL aliquot was mixed with a methanol solution (400 μL, Cat. No. CAEQ-4-000306-4000, CNW Technologies) and acetonitrile (Cat. No. CAEQ-4-000308-4000, CNW Technologies) in a 1:1 ratio. The resulting mixture was subjected to centrifugation at 12,000 rpm for 15 min at 4 °C. The supernatant was subsequently collected for further UHPLC-MS/MS analysis. A 1 mg/mL stock solution of the standard was prepared and subsequently diluted to generate a series of calibration solutions. For UHPLC-PRM-MS analysis, the target compounds underwent chromatographic separation utilizing a Vanquish UPLC system (Thermo Fisher Scientific) equipped with a Waters ACQUITY UPLC BEH C18 column (150 mm × 2.1 mm, 1.7 μm, Waters). The mobile phase for liquid chromatography comprised a 5 mmol/L aqueous ammonium acetate solution (Phase A, catalog number CAEQ-4-013465-0100, CNW Technologies) and acetonitrile (Phase B). The column compartment was maintained at 45°C and the sample tray at 4 °C, with a 1 μL injection volume. Mass spectrometric analysis was performed with an Orbitrap Exploris 120 high-resolution mass spectrometer operating in parallel reaction monitoring (PRM) mode.

#### 2.4.5 Real-time quantitative PCR (RT-qPCR) analysis

Total RNA was extracted from rat myocardial tissue using an ultrapure RNA extraction kit. RNA purity and concentration were evaluated, and then reverse transcription was carried out to synthesize complementary DNA (cDNA). The PCR reaction was conducted in a 20 μL volume, beginning with an initial denaturation at 95 °C for 10 min, followed by 43 amplification cycles, each consisting of 10 s at 95 °C for denaturation, 10 s at 60 °C for primer annealing, and 10 s at 72 °C for extension. Gene expression levels were quantified using the 2^–ΔΔ^*^Ct^* method, with GAPDH serving as the internal control. Primers were designed and synthesized by Hunan Accurate Biology, with sequences detailed in Table 1.

**TABLE 1:** Primer sequences for real-time quantitative PCR.

Target gene		Primer sequence(5′→3′)
TGR5	Forward	CTATGGAATAGGAGCCATCAGGG
	Reverse	GCAGGGAGAGGAAACAAAAGTTG
GAPDH	Forward	GGCACAGTCAAGGCTGAGAATG
	Reverse	ATGGTGGTGAAGACGCCAGTA

#### 2.4.6 Western blot analysis

Cardiac tissues and cells were collected in EP tubes and lysed with RIPA buffer. After homogenization and a 30-minute incubation on ice, samples were centrifuged at 12,000 rpm for 10 min at 4 °C. Supernatants were collected for protein quantification using the BCA assay. Equal amounts of protein from each group were separated electrophoretically using a fast gel kit. The proteins were transferred onto PVDF membranes, and then blocked with a 5% milk solution for 2 h. Membranes were incubated overnight with TGR5 and GAPDH antibodies, followed by incubation with a secondary antibody at 37 °C for 2 h. Protein signals were analyzed using “lmedium” software. Immunoblot analysis was performed using the following antibodies: TGR5 (1: 1000, Abcam, ab72608), GAPDH (1: 1000, Servicebio, GB11002), and horseradish peroxidase-goat anti-rabbit (1:3000, Servicebio, GB23303).

#### 2.4.7 ELISA assay of BNP, cAMP, IL-6, TNF-α, IL-1β

Serum samples from rats and the supernatants of cultured cells were collected for analysis. The concentrations of serum cAMP, BNP and inflammatory cytokines interleukin-1 beta (IL-1β), IL-6, and tumor necrosis factor-alpha (TNF-α) in both serum and supernatants were quantified using commercially available ELISA assay kits: for cAMP (Cat No. A107988,Shanghai Fusheng Industrial Co., Ltd., China), BNP (Cat No. AF2943-A, Jiangsu Jingmei Biotechnology Co., Ltd., China), IL-1β (Cat No. AF2923-A, Jiangsu Jingmei Biotechnology Co., Ltd., China), IL-6 (Cat No. AF3066-A, Jiangsu Jingmei Biotechnology Co., Ltd., China) and TNF-α (Cat No. AF3056-A, Jiangsu Jingmei Biotechnology Co., Ltd., China).

### 2.5 Statistical analysis

Data were statistically analyzed using one-way ANOVA or, for non-normally distributed data, the Kruskal-Wallis test, utilizing SPSS 26.0. The Spearman’s Rank Correlation Coefficient method was employed to visualize the correlations among the data. A *P*-value of <0.05 was considered statistically significant, whereas *P*-value of <0.01 indicated high significance. Data distributions were visualized by generating histograms using GraphPad Prism 9.0.

## 3 Results

### 3.1 Chemical composition and stability of YXT

**FIGURE 1:**
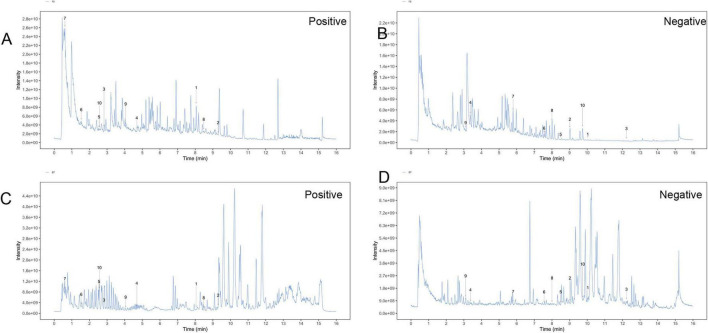
Total ion chromatograms (TIC) of YXT by UHPLC-OE-MS. **(A)** TIC of YXT samples in positive ion mode; **(B)** TIC of YXT samples in negative ion mode; **(C)** TIC of compound sample of YXT serum samples in positive ion mode; **(D)** TIC of compound sample of YXT serum samples in negative ion mode.

To clarify the mechanism through which YXT treats CHF, we analyzed the blood entry chemistry of YXT using UHPLC-QE/MS and identified 1049 blood entry chemistry components, including saponins, flavonoids, sterols, phenolic compounds, phenolic acids, terpenoids, and other related compounds, compounds such as 3-O-Acetyl-16alpha-hydroxytrametenolic acid, Alisol A, Alisol F, Foliosidine, Pachymic acid, Pygenic acid A and B, Formononetin, 29-hydroxypolyporenic acid C, Astragaloside IV, Kaempferol 7-O-glucoside, Ginsenoside Rg3, Tanshinone IIb, Rosmarinic acid, Ginsenoside Rg5, o-Xylene, Formononetin 7-(6”-malonylglucoside), Microtoenin B, and Cryptotanshinone, among others, are included (Figure 1 and Tables 2, 3).

**TABLE 2:** Chemical composition list of YXT in positive ion mode.

ID	Name EN	Formula	mzmed	rtmed	Type
1	Alisol F	C30H48O5	511.3406158	483.4705	Pos
2	Cryptotanshinone	C19H20O3	297.1483005	557.9945	Pos
3	Guanosine	C10H13N5O5	284.1000727	169.322	Pos
4	Salviamine B	C18H13NO3	292.0964186	281.054	Pos
5	Isorhamnetin	C16H12O7	317.0649346	152.402	Pos
6	Kaempferol 7-O-glucoside	C21H20O11	449.1075539	92.13285	Pos
7	p-Coumaric acid	C9H8O3	165.0543267	36.27725	Pos
8	Tanshinone IIb	C19H18O4	311.1278585	508.9575	Pos
9	Formononetin 7-(6”-malonylglucoside)	C25H24O12	517.1336145	243.029	Pos
10	Methyl cinnamate	C10H10O2	163.0751635	153.482	Pos

**TABLE 3:** Chemical composition list of YXT in negative ion mode.

ID	Name EN	Formula	mzmed	rtmed	Type
1	3-O-Acetyl-16alpha-hydroxytrametenolic acid	C32H50O5	513.3580238	601.897	Neg
2	Alisol A	C30H50O5	535.3641607	540.952	Neg
3	Pachymic acid	C33H52O5	527.3741541	733.7295	Neg
4	Formononetin	C16H12O4	267.0664777	202.63	Neg
5	29-Hydroxypolyporenic acid C	C31H46O5	497.3275221	509.751	Neg
6	Fargoside E	C42H66O14	793.4399862	453.367	Neg
7	Astragaloside IV	C41H68O14	829.4583253	347.675	Neg
8	Ginsenoside Rg3	C42H72O13	783.4894956	480.099	Neg
9	Rosmarinic acid	C18H16O8	359.0779182	185.834	Neg
10	Ginsenoside Rg5	C42H70O12	765.4783791	583.9125	Neg

### 3.2 YXT improves cardiac function in CHF rats

**FIGURE 2:**
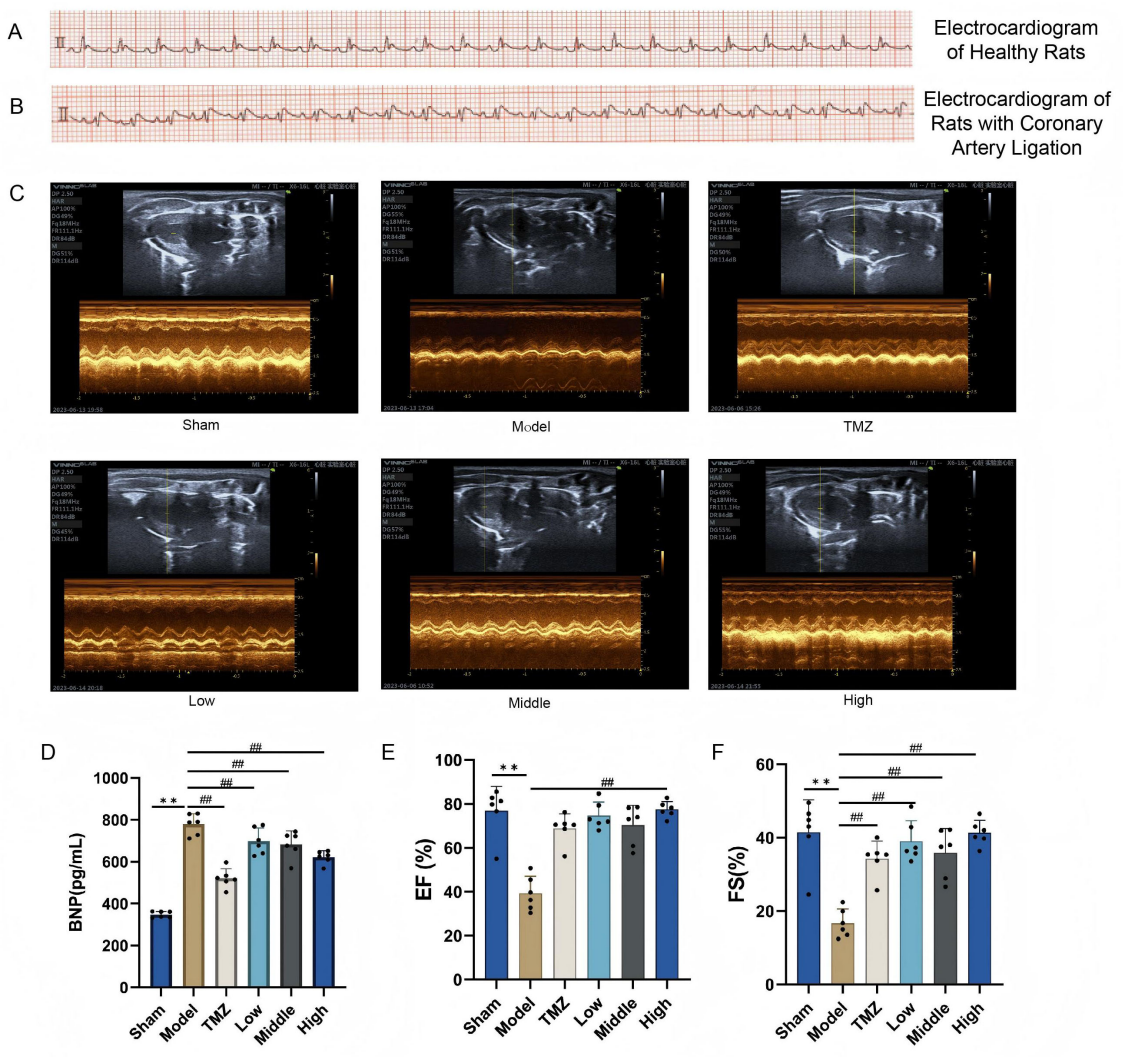
Cardiac ultrasound results and serum BNP levels in rats. **(A)** Electrocardiograms of healthy rats; **(B)** Coronary artery ligation-induced heart failure rats; **(C)** Quantitative evaluation of cardiac function based on short axis images of 2 D echocardiography; **(D)** Serum BNP levels; **(E)** EF was used to determine cardiac systolic function; **(F)** FS was used to determine cardiac systolic function. **P < 0.01, vs Sham; ^##^*P* < 0.01, vs Model; all values are expressed as the SD ± mean.

Electrocardiographic analysis revealed normal waveforms in healthy rats, whereas ligated rats exhibited significant ST-segment elevation and peaked T-waves, confirming successful coronary ligation and myocardial ischemia. The EF and FS value significantly decreased and the BNP level significantly increased in the Sham group (*P* < 0.01). After treatment, TMZ and YXT at all doses reduced serum BNP levels (*P* < 0.01), the EF value of High group, FS of TMZ and YXT at all doses increased (*P* < 0.01), (Figure 2).

### 3.3 YXT improves the pathological damage of heart tissue and colon in CHF rats

**FIGURE 3:**
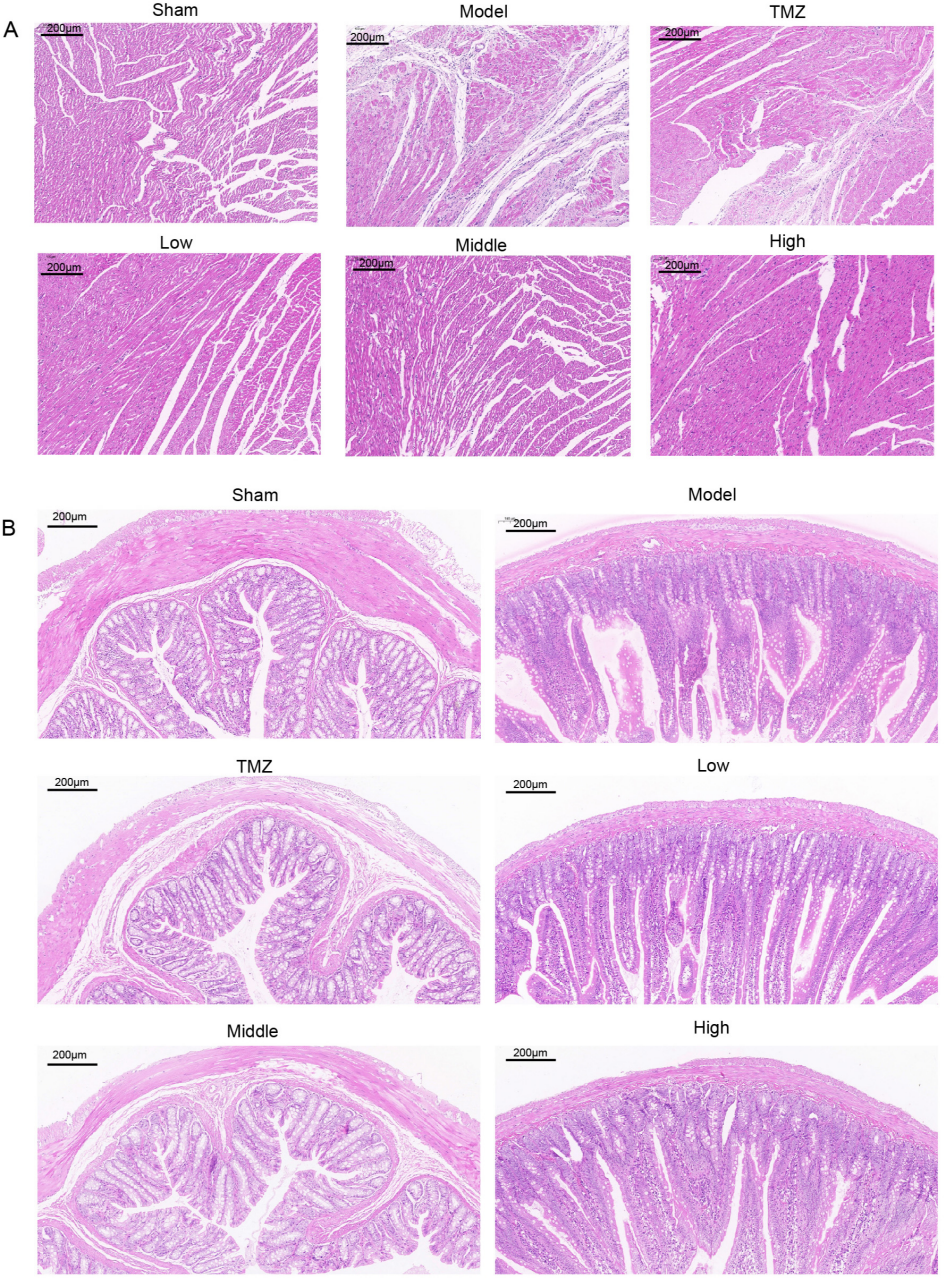
Representative histopathological images of rat heart and colon tissues at 100x magnification: **(A)** Representative HE stained images of heart tissue; **(B)** Representative HE stained image of colonic tissue.

The histological results in both cardiac and colonic tissues are depicted in Figure 3. HE staining revealed that the cardiac tissue of the Sham group displayed a well-organized arrangement of cardiomyocytes without significant pathological changes. Conversely, the Model group exhibited substantial morphological alterations, such as distorted cells, edema, disorganized structure, and epicardial infiltration. Following treatment, the TMZ, Low, Middle, and High groups demonstrated varying degrees of improvement in cardiac tissue integrity compared with the Model group. Meanwhile, the colonic tissue of the Sham group displayed no significant inflammatory infiltration; goblet cells were regularly arranged, the number of glands remained unchanged, and the crypt structure was well-defined. Conversely, in the Model group, rats demonstrated a reduction in the number of goblet cells with disordered arrangement, marked inflammatory infiltration in multiple regions, and an indistinct crypt structure. Following treatment, the pathological alterations in the colonic structure of the rats were mitigated, with the most pronounced improvements observed in the TMZ group and the Middle-dose YXT group.

### 3.4 YXT improves the gut microbiota disorder in CHF rats

**FIGURE 4:**
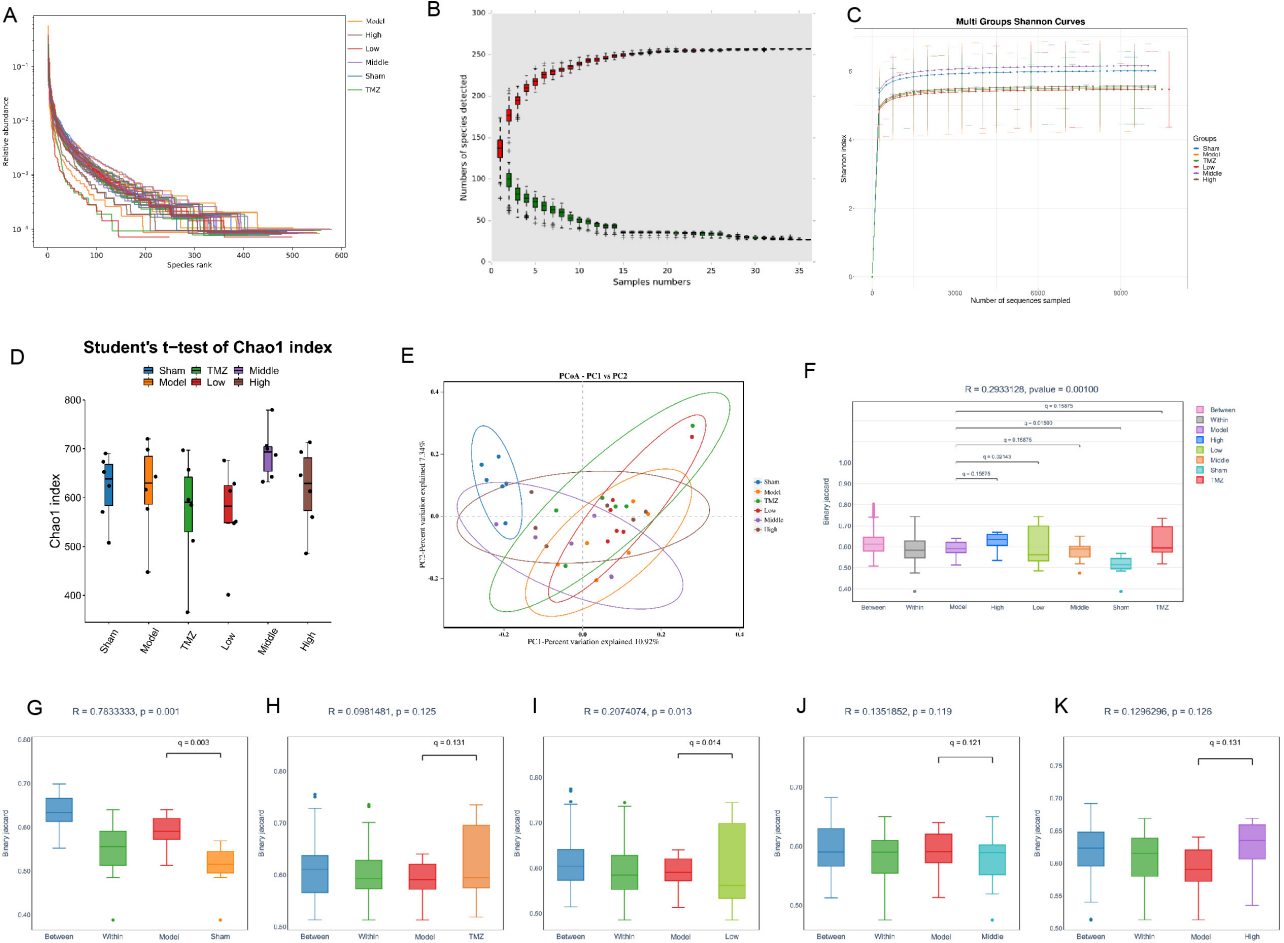
Analysis of the diversity gut microbiota. **(A)** Sample correlation coefficient curve; **(B)** Species accumulation curve; **(C)** Shannon curve item index curve; **(D)** Chao1 index; **(E)** PCoA analysis; **(F)** Anosim analysis boxplot; **(G)** Anosim analysis between the Model and Sham groups; **(H)** Anosim analysis between the Model and TMZ groups; **(I)** Anosim analysis between the Model and Low groups; **(J)** Anosim analysis between the Model and Middle groups; **(K)** Anosim analysis between the Model and High groups.

The gut microbiome plays a critical role in the development and progression of CHF. Therefore, we analyzed 16S rRNA gene sequences from fecal samples of the colon to evaluate the structural changes in the gut microbial community. As shown in Figures 4A–C, the correlation coefficient curve, species accumulation curve, and Shannon diversity index for all experimental groups exhibited a trend toward stabilization, reflecting a rich and uniform gut microbiota. Moreover, the stabilization of these metrics suggests that the sequencing depth was sufficient to accurately reflect the complexity and diversity of the microbial communities under investigation. Figures 4D–F reveal a reduction in the Chao1 in the Model group compared with the Sham group. Figures 4G–K shows that inter-group distances exceeded intra-group distances, with a significant difference between the Model and Sham groups. After treatment, the distances between the TMZ, Low, Middle, High groups and the Model were notably larger than within each group, especially in the Low group, where differences were statistically significant. However, these indices increased after TMZ and YXT treatments, with the Middle group showing the most significant improvement. Principal Coordinates Analysis (PCoA) and anosim analysis was conducted to assess beta diversity and revealed that distances within groups were smaller than those between groups. Additionally, the Model and Sham groups were clearly distinct, suggesting notable differences in microbiota diversity. Collectively, these findings indicate that YXT treatment may significantly improve gut microbiota diversity in CHF rats.

**FIGURE 5:**
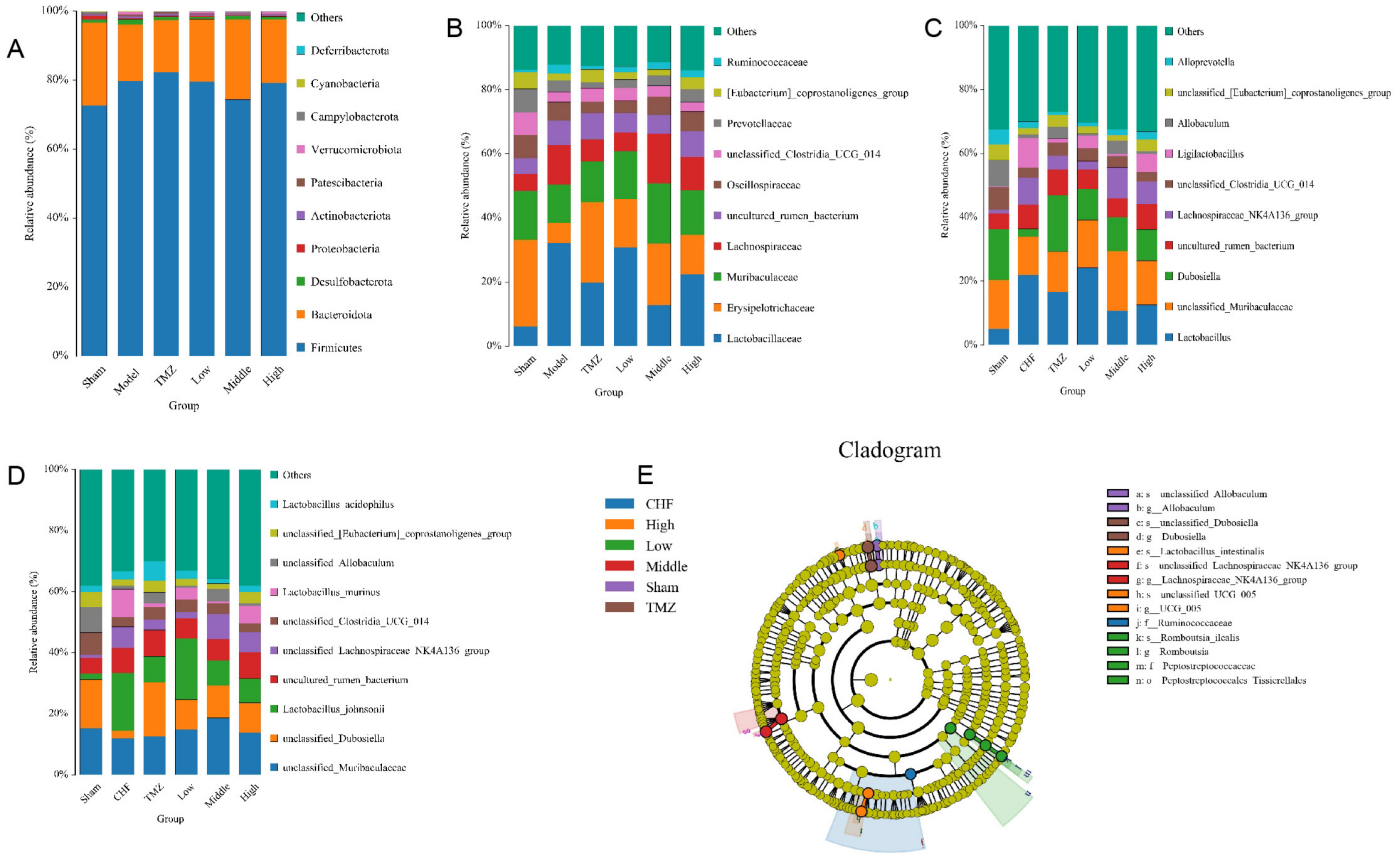
Analysis of the abundance of gut microbiota. **(A)** Gut microbiota relative abundance at phylum level; **(B)** Gut microbiota relative abundance at family level; **(C)** Gut microbiota relative abundance at genus level; **(D)** Gut microbiota relative abundance at species level; **(E)** LEfSe evolutionary branching diagram.

Relative to Sham group, the Model group showed a marked elevation abundances of Firmicutes, Desulfobacterota, Actinobacteriota, Patescibacteria, Verrucomicrobiota, and Cyanobacteria at the phylum level. However, Bacteroidota, Proteobacteria, and Campylobacterota were substantially depleted. The abundance of *Firmicutes, Desulfobacterota, Actinobacteriota, Patescibacteria, Verrucomicrobiota*, and *Cyanobacteria* decreased, whereas that of *Bacteroidota* and *Campylobacterota* bacteria increased in the TMZ, Low, Middle, and High groups versus the Model group (Figure 5A). At the family level, the Model group displayed higher relative abundances of *Lactobacillaceae, Lachnospiraceae, Ruminococcaceae*, and an *uncultured rum bacterium* compared with the Sham group. Conversely, the relative abundances of *Erysipelotrichaceae, Muribaculaceae, Oscillospiraceae*, unclassified Clostridia UCG-014, *Prevotellaceae*, and the *[Eubacterium] coprostanoligenes* group were diminished in the Model group compared with the Sham group. The relative abundances of *Lactobacillaceae*, *Lachnospiraceae*, and *Ruminococcaceae* were decreased, while those of *Erysipelotrichaceae*, *Muribaculaceae*, *Oscillospiraceae*, *Prevotellaceae*, and the *[Eubacterium] coprostanoligenes* group were augmented in the TMZ, Low, Middle, and High groups compared with the Model group. Additionally, the relative abundance of *unclassified Clostridia UCG-014* was elevated in the TMZ, Low, and Middle groups (Figure 5B). At the genus level, the Model group showed increased abundances of *Lactobacillus*, *an unclassified rum bacterium*, *Lachnospiraceae_NK4A136_group*, and *Ligilactobacillus* compared with the Sham group. Conversely, the abundance ratios of unclassified *Muribaculaceae*, *Dubosiella*, *unclassified Clostridia_UCG_014*, *Allobaculum*, unclassified *[Eubacterium]_coprostanoligenes_group*, and *Alloprevotella* were reduced in the Model group compared with the Sham group. The abundance proportions of *Lactobacillus*, *Lachnospiraceae*_*NK4A136_group*, and *Ligilactobacillus* decreased, whereas the abundance ratios of *unclassified Clostridia_UCG_014*, *unclassified Muribaculaceae, Dubosiella*, and unclassified [*Eubacterium]_coprostanoligenes_group* increased in the TMZ, Low, Middle, and High groups compared with the Model group (Figure 5C). At the species level, it was found that the abundances of uncultured rum bacterium and unclassified_*Lachnospiraceae_NK4A136_group* were significantly higher, while the abundance ratios of *unclassified_Muribaculaceae*, *unclassified_Dubosiella, Lactobacillus_johnsonii*, *unclassified_ Clostridia_UCG_014*, *unclassified_Allobaculum*, and *unclassified_ [Eubacterium]coprostanoligenes_group* were notably lower in the Model group than in the Sham group. In addition, the abundance ratios of *unclassified_Lachnospiraceae_NK4A136_group*, *Lacto bacillus_murinus*, and *Lactobacillus_acidophilus* were decreased, whereas the proportions of *unclassified_Muribaculaceae*, *unclassified_Dubosiella*, and *unclassified[Eubacterium]_coprostanoligenes_group* were increased in the TMZ, Low, Middle, and High groups compared with the Model group (Figures 5D, E).

### 3.5 YXT improves serum BA levels in CHF rats

**FIGURE 6:**
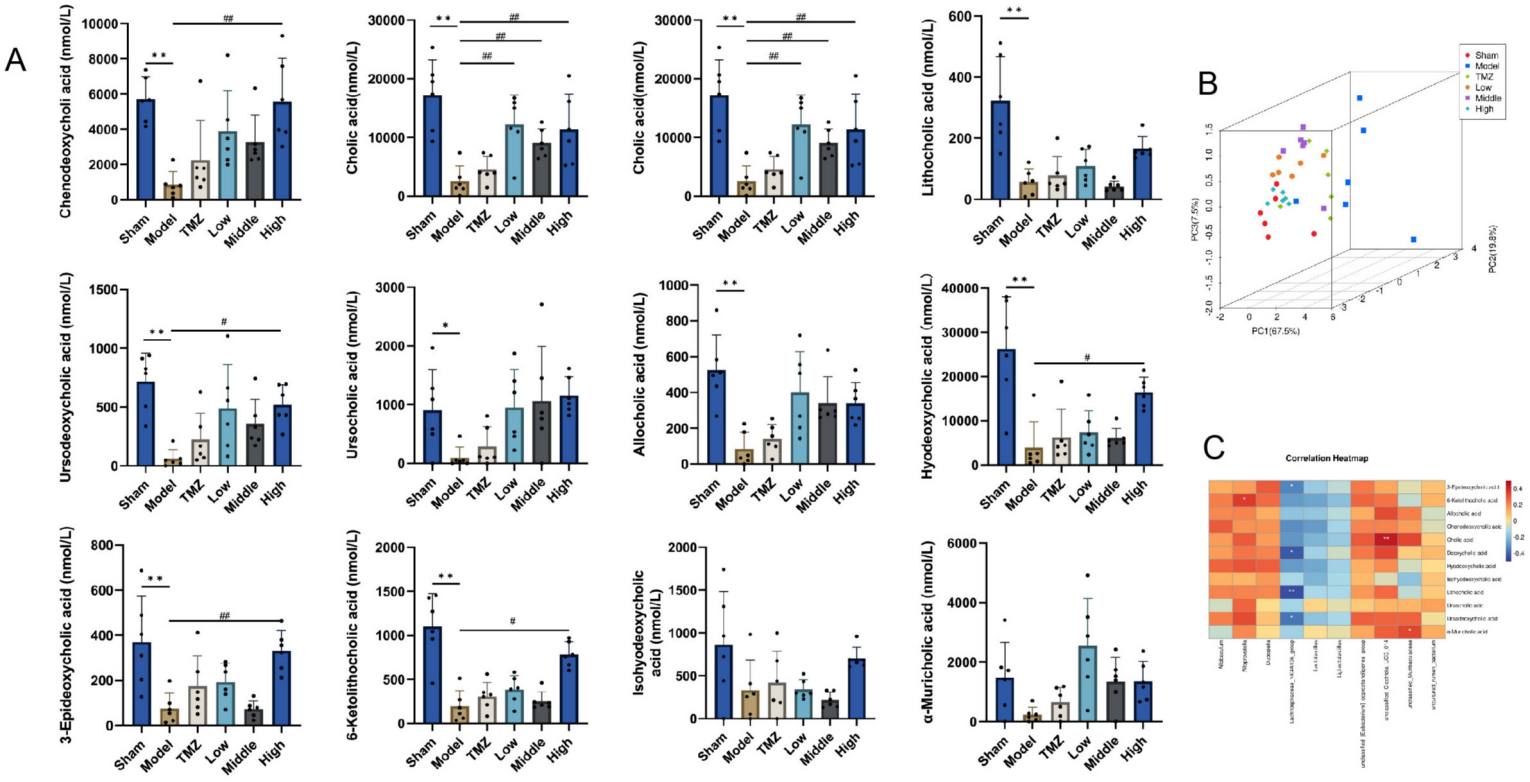
Serum BA levels in rat and correlation analysis. **(A)** Serum bile acid levels in rats; **(B)** PCA score plot 3D; **(C)** Correlation analysis of gut microbiota and BA at genus level; **P < 0.01, *P < 0.05 vs Sham; ^##^*P* < 0.01, ^#^*P* < 0.05 vs Model, data are expressed as the mean ± SD values.

The gut microbiota significantly influences BA metabolism through BA hydrolysis enzymes, affecting BA biosynthesis and their corresponding signaling pathways. Hence, we investigated the relationship between gut microbiota at the genus level and BA to elucidate the intricate interactions between microbial populations and BA. In comparison to the Sham group, the Model group demonstrated a significant reduction in serum levels of various BA, including CDCA, CA, DCA, LCA, ursodeoxycholic acid (UDCA), ursocholic acid (UCA), allocholic acid (ACA), hyodeoxycholic acid (HDCA), 3-epideoxycholic acid (3-EDCA), 6-ketolithocholic acid (6-KLCA), isohyodeoxycholic acid (IsoHDCA), and α-muricholic acid (α-MCA), with statistical significance (*P* < 0.05 or *P* < 0.01). Subsequent to treatment, an elevation in these BA levels was observed across the TMZ, Low-, Middle-, and High-dose Yixintai groups. Notably, the High-dose group exhibited statistically significant increases in CDCA, CA, DCA, UDCA, HDCA, 3-EDCA, and 6-KLCA (*P* < 0.05 or *P* < 0.01). Additionally, the Low- and Middle-dose groups showed significant upregulation of CA (*P* < 0.05 or *P* < 0.01). The results are illustrated in the heatmap shown in Figure 6. It was found that certain bacteria, such as *Allobaculum*?*Alloprevotella*, *Dubosiella*, *unclassified_[Eubacterium]_coprostanoligenes_group*, *Clostridia_UCG_014*, *unclassified_Muribaculaceae* and *uncultured_rumen_bacterium* were positively correlated with nearly all BA. However, *Lachnospiraceae_NK4A136_group*, *Lactobacillus* and *Ligilactobacillus* exhibited were negatively correlated with most BA.

### 3.6 YXT improves the expression of TGR5 and suppresses the cardiac inflammatory response

**FIGURE 7:**
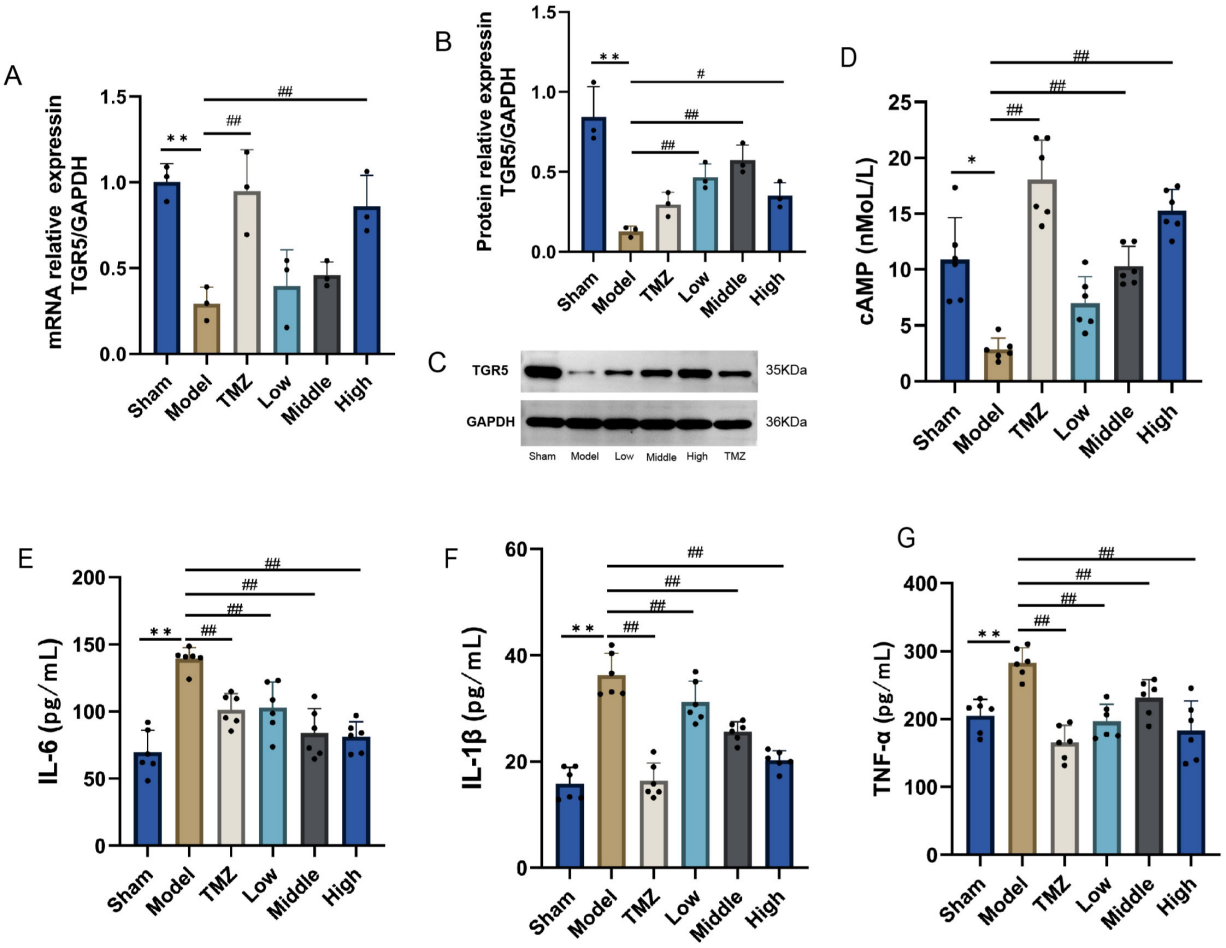
TGR5, cAMP expression levels and inflammatory factor levels in CHF rats heart tissue. **(A)** TGR5 mRNA expression; **(B)** Relative protein expression of TGR5; **(C)** TGR5 Western Blot; **(D)** Serum cAMP; **(E)** Serum IL-1 β; **(F)** Serum IL-6; **(G)** Serum TNF- α. **P < 0.01, *P < 0.05 vs Sham; ^##^*P* < 0.01, ^#^*P* < 0.05 vs Model, data are expressed as the mean ± SD values.

As depicted in Figure 7, mRNA and protein expression levels of TGR5 were notably reduced in the Model group when compared with the Sham group (*P* < 0.01), post-treatment by TMZ and YXT, the mRNA and protein expression levels of TGR5 were markedly elevated relative to the Model group (*P* < 0.05 or *P* < 0.01), the serum concentrations of cAMP were lower in the Model group (*P* < 0.05), after treatment, the serum cAMP levels were significantly elevated in the TMZ, Middle and High groups when compared to the Model group (*P* < 0.01). The serum concentrations of, IL-1β, IL-6, and TNF-α were notably higher in the Model group. However, after treatment, these serum levels were significantly reduced in the TMZ, Low, Middle, and High groups when compared to the Model group. These results suggest that YXT can inhibit the inflammatory response in heart failure rats by activating TGR5 expression.

### 3.7 YXT drug-containing serum promotes the expression of TGR5 in H9c2 cells and improves the inflammatory damage of H9c2 cells

**FIGURE 8:**
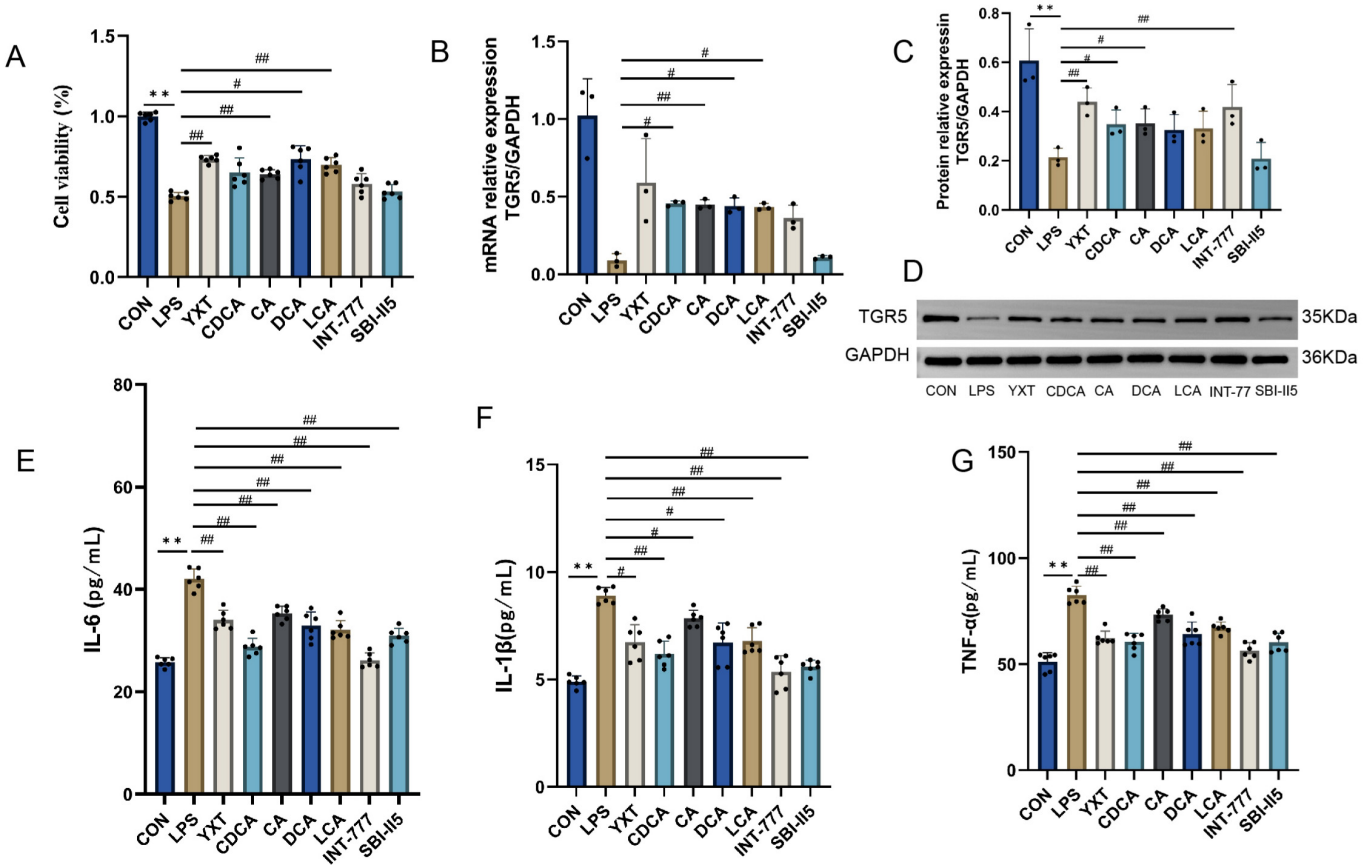
H9c2 cell viability, TGR5, cAMP expression levels, and inflammatory factor levels. **(A)** The viability in the indicated groups; **(B)** TGR5 mRNA expression; **(C)** Relative protein expression of TGR5; **(D)** TGR5 Western Blot; **(E)** IL-16 content in the cell supernatant; **(F)** IL-β content in the cell supernatant; **(G)** TNF-α level in cell supernatant **P < 0.01, *P < 0.05 vs CON group; ##*P* < 0.01, #*P* < 0.05 vs LPS group, Data are expressed as the mean ± SD values.

As illustrated in Figure 8, cell viability was significantly inhibited after LPS intervention in H9c2 cardiomyocytes compared with the CON group (*P* < 0.01). Compared with the LPS group, the cell viability was markedly increased after treatment with YXT containing serum, CA, DCA, and LCA (*P* < 0.01). The mRNA levels of TGR5 in H9c2 cells were elevated in the CDCA, CA, DCA, and LCA groups compared with the LPS group (*P* < 0.05 or *P* < 0.01). Compared with the CON group, the LPS group exhibited a downward trend in the TGR5 protein levels (*P* < 0.01), the protein levels of TGR5 in H9c2 cells from the YXT, CDCA, CA, and INT-777 groups were increased (*P* < 0.05 or *P* < 0.01). Compared with the CON group, the supernatant levels of IL-6, IL-1β and TNF-α in H9c2 cells were significantly higher in the LPS group (*P* < 0.01), treatment with YXT, CDCA, CA, DCA, LCA, and INT-777 significantly reduced IL-6, IL-1β and TNF-α levels in H9c2 cell supernatants (*P* < 0.05 or *P* < 0.01). The results suggest that the activation of TGR5 plays a mediating role in the anti-inflammatory effects of Yixintai on H9c2 cells, as demonstrated by the observed reduction in inflammatory injury following treatment with serum containing the drug.

## 4 Discussion

Chronic heart failure (CHF) is a complex clinical condition influenced by multiple factors such as congenital heart defects, diabetes, obesity, smoking, myocarditis, cardiomyopathy, hypertension, and coronary artery disease. Therefore, CHF should be viewed as a multifaceted syndrome rather than a singular disease (Baman and Ahmad, 2020). The gut microbiota, as the largest microbial ecosystem in the human body, plays a crucial role in regulating a range of physiological functions. Maintaining the dynamic balance of this microbiota is crucial for human health, the formation of a mucosal immune barrier, and the prevention of pathogen colonization (Branchereau et al., 2019). During CHF, reduced cardiac EF and output cause both systemic and gastrointestinal congestion, leading to ischemia, hypoxia, and edema in the intestinal mucosa, thereby compromising the intestinal barrier and disrupting microcirculation. Concurrently, the equilibrium of gut bacteria is disrupted, promoting bacterial translocation, endotoxemia, and the secretion of inflammatory mediators. These processes initiate systemic inflammation, impair vascular endothelial function, impede blood flow and nutrient delivery, and ultimately result in multi-organ dysfunction, thereby exacerbating the severity of CHF (Formiga et al., 2019; Jain et al., 2024; Yang et al., 2024). The present study demonstrated that CHF rats showed substantial infiltration of inflammatory cells and disruption of intestinal mucosal integrity, suggesting an inflammatory response. Elevated serum levels of IL-1β, IL-6, TNF-α, and BNP, along with reduced EF and impaired cardiac function, corroborated these findings. Conversely, rats treated with YXT showed a significant reduction in serum inflammatory markers, enhanced cardiac function, and alleviated pathological changes in both cardiac and colonic tissues. These findings indicate that YXT effectively alleviates CHF by reducing the inflammatory response.

Patients with CHF exhibited reduced gut microbiota diversity, increased abundance of harmful bacteria like *Shigella*, *Campylobacter, Salmonella, Yersinia*, and various *Candida* species. In contrast, they have lower number of beneficial bacteria, such as *Bifidobacterium* and *Lactobacillus* (Pasini et al., 2016). The severity of CHF is influenced by certain pathogens, with *Shigella*, *Campylobacter*, and *Candida*, considered as important microbiota affecting the prognosis (Yuzefpolskaya et al., 2020). Research suggests that disruption of gut microbiota may trigger chronic inflammation and immune dysfunction, potentially aggravating HF by lowering ejection fraction (Madan and Mehra, 2020). In this study, we found that rats with CHF exhibited reduced microbial diversity. However, the YXT treatment restored the normal diversity of gut microbiota, supporting the notion that YXT can enhance cardiac function in CHF rats by rebalancing gut microbiota (Cui et al., 2018). Compared to the Sham group, the Model group exhibited an elevated *Firmicutes* abundance but a reduced *Bacteroidota* level. YXT administration significantly reversed these microbial shifts, lowering *Firmicutes* and enhancing *Bacteroidota* populations. This observation aligns with prior reports by Li et al. (2021); Zhang Q. L. et al. (2023) on gut microbiota modulation. In the study undertaken by Oliphant et al. (2021), Bacteroides was an important constituent of the microbiota’s bacterial community. Furthermore, studies have demonstrated that *Bacteroidota* plays a pivotal role in generating health-promoting metabolites and modulating host immune responses. Furthermore, Zhang et al. (2020) demonstrated that *Desulfobacterota* contributes to intestinal inflammation, suggesting that fluctuations in their abundance were associated with LPS levels. Recent studies have identified specific microbiota associated with systemic inflammation. For instance, Liu et al. (2022) demonstrated a direct link between *Actinobacteria* and inflammatory processes, suggesting that elevated abundance of Actinobacteria may trigger an inflammatory cascade. Similarly, Drapkina et al. (2022) and her team reported that HF patients with reduced ejection fraction had elevated levels of *Lactobacillaceae* levels, which correlated with a more pronounced systemic inflammatory response. Elsewhere, Li et al. (2024). postulated that *Lachnospiraceae* could be a pathogenic factor, exacerbating the occurrence of inflammatory reactions. In this study, it was observed that rats with CHF exhibited higher abundances of *Desulfobacterota, Actinobacteriota, Lactobacillaceae*, and *Lachnospiraceae*. Following treatment with YXT, a notable reduction in the abundance of these specific microbiota was observed. This observation implies that YXT might contribute to alleviate the abundance of pathogenic bacteria in rats suffering from CHF.

Gut microbiota are important regulators of the cardiovascular health, with studies showing that compromised intestinal barrier will lead to bacterial translocation, causing activation of inflammatory and immune pathways. These mechanisms are implicated in the synthesis of various metabolites, including BA, SCFAs, TMAO, and other microbial byproducts. BA improve nutrient uptake in the intestines, regulating lipid and energy metabolism, and sustaining intestinal balance. Emerging evidence show that alterations in BA metabolic pathways may provoke inflammatory responses, thereby increasing the risk of developing CHF (Zhang S. et al., 2023). A prospective cohort study by Mayerhofer et al. reported that the BA levels were reduced in patients with CHF (Mayerhofer et al., 2017). Moreover, the study revealed that the serum BA levels in rats with CHF were decreased, but restored following treatment. Other investigations have shown that *Actinobacteria* and *Proteobacteria* are involved in modulating the activity of BA, including chenodeoxycholic acid, cholic acid, deoxycholic acid, and lithocholic acid, which are essential for maintaining BA balance. Through receptor-mediated mechanisms, BA not only shape the gut microbiota but also orchestrate host metabolic and inflammatory processes (Komorniak et al., 2024). Herein, the Pearson’s correlation analysis demonstrated a strong correlation between the abundance of specific gut microbiota, including *[Eubacterium]_coprostanoligenes_group*, *Allobaculum, Alloprevotella*, *Clostridia_UCG_014, Dubosiella*, *uncultured_rumen_bacterium*, and *Muribaculaceae*, and bile acid (BA) levels. This suggests that these microbial communities may increase the production of BA by modulating the activity of bile salt hydrolase (BSH). Therefore, YXT may regulate the abundance of *[Eubacterium]_coprostanoligenes_group, Allobaculum, Alloprevotella, Clostridia_UCG_014, Dubosiella, uncultured_rumen_bacterium* and *Muribaculaceae* to augment BA metabolism and mitigate the inflammatory response associated with CHF.

Following their synthesis in the liver, BA are transported to the intestine where they act as molecular signals regulating cardiovascular function. TGR5, a widely researched membrane-bound G protein-coupled receptor, is expressed in multiple tissues throughout the body (Wahlström et al., 2016; Winston and Theriot, 2020; Perino et al., 2021). Prior investigations show that BA activate the TGR5 receptor to improve the function of cardiomyocytes, enhancing diastolic function, reducing inflammation in HF, and stimulating the restoration of intestinal barrier (Liu et al., 2013; Zuo et al., 2015; Hanafi et al., 2018). Moreover, experimental evidence indicates that BA, functioning as TGR5 agonists, can protect murine myocardium, improving the heart’s ability to respond to physiological, muscular, and hemodynamic stress. Considering that TGR5 participates in myocardial adaptability, its activation may be an attractive approach to treat HF (Eblimit et al., 2018). Moreover, disturbances in the gut microbiome and BA have been associated with the development of HF. BA activates the TGR5, thereby inhibiting inflammatory responses. This mechanism highlights the role of BA in immune regulation (Chen et al., 2020; Hu et al., 2021). In rats with CHF, TGR5 and cAMP expression levels were decreased, while serum levels of IL-1β, IL-6, and TNF-α were markedly increased. Interestingly, YXT treatment restored TGR5 and cAMP levels in cardiac tissues and reduced serum concentrations of IL-1β, IL-6, and TNF-α. These results suggest that YXT upregulates TGR5 expression in the heart to mitigate the inflammatory response in CHF rats. *In vitro* experiments showed that YXT-containing serum, combined with LCA, DCA, CDCA, CA, and the TGR5 agonist INT-777, alleviated LPS-induced damage in H9c2 cells. This treatment stimulated TGR5 and cAMP expression, suppressed inflammatory response and improved cardiomyocyte survival. Collectively, these findings indicate that the YXT’s protective effects against LPS-induced H9c2 cardiomyocyte injury model were mediated by the interaction of BA with the TGR5 signaling pathway.

**FIGURE 9:**
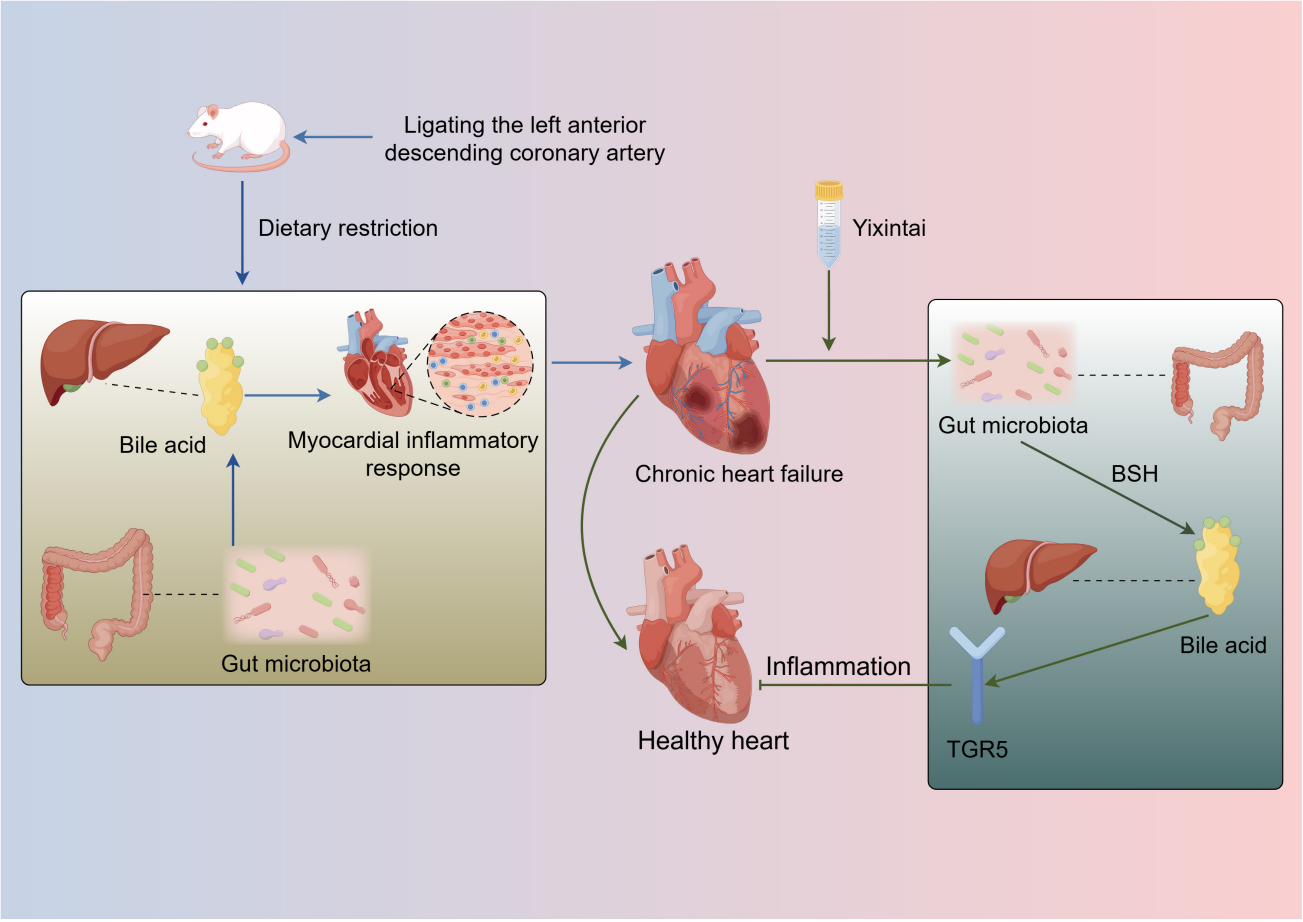
The mechanism YXT regulates gut microbiota and bile acid to treat CHF.

In conclusion, this study illustrates that YXT ameliorates gut microbiota dysbiosis in rats with CHF, subsequently modulating serum BA levels and activating the BA receptor TGR5 to suppress inflammatory responses, thereby achieving therapeutic effects against CHF. Nonetheless, several limitations must be acknowledged. While differential microbial taxa and BA levels were identified, further research is necessary to comprehensively elucidate the underlying mechanisms, future studies should investigate these findings in greater depth (Figure 9).

## Data Availability

The original contributions presented in the study are publicly available. This data can be found here: https://www.ncbi.nlm.nih.gov/bioproject/PRJNA1344468.

## References

[B1] Baman J. R. Ahmad F. S. (2020). Heart failure. *JAMA* 324:1015. 10.1001/jama.2020.13310 32749448

[B2] Bonfioli G. B. Rodella L. Metra M. Vizzardi E. (2025). GLP-1 receptor agonists as promising anti-inflammatory agents in heart failure with preserved ejection fraction. *Heart Fail. Rev.* 30 131–136. 10.1007/s10741-024-10450-6 39425816 PMC11646221

[B3] Branchereau M. Burcelin R. Heymes C. (2019). The gut microbiome and heart failure: A better gut for a better heart. *Rev. Endocr. Metab. Disord.* 20 407–414. 10.1007/s11154-019-09519-7 31705258

[B4] Chen W. Wei Y. Xiong A. Li Y. Guan H. Wang Q. (2020). Comprehensive analysis of serum and fecal bile acid profiles and interaction with gut microbiota in primary biliary cholangitis. *Clin. Rev. Allergy Immunol.* 58 25–38. 10.1007/s12016-019-08731-2 30900136

[B5] Cui X. Ye L. Li J. Jin L. Wang W. Li S. (2018). Metagenomic and metabolomic analyses unveil dysbiosis of gut microbiota in chronic heart failure patients. *Sci. Rep.* 8:635. 10.1038/s41598-017-18756-2 29330424 PMC5766622

[B6] Drapkina O. M. Ashniev G. A. Zlobovskaya O. A. Yafarova A. A. Dementeva E. V. Kaburova A. N. (2022). Diversities in the gut microbial patterns in patients with atherosclerotic cardiovascular diseases and certain heart failure phenotypes. *Biomedicines* 10:2762. 10.3390/biomedicines10112762 36359282 PMC9687836

[B7] Eblimit Z. Thevananther S. Karpen S. J. Taegtmeyer H. Moore D. D. Adorini L. (2018). TGR5 activation induces cytoprotective changes in the heart and improves myocardial adaptability to physiologic, inotropic, and pressure-induced stress in mice. *Cardiovasc. Ther.* 36:e12462. 10.1111/1755-5922.12462 30070769 PMC6800140

[B8] Formiga F. Ferreira Teles C. I. Chivite D. (2019). Impact of intestinal microbiota in patients with heart failure: A systematic review. *Med. Clin.* 153 402–409. 10.1016/j.medcli.2019.06.006 31416611

[B9] Hanafi N. I. Mohamed A. S. Sheikh Abdul, Kadir S. H. Othman M. (2018). Overview of bile acids signaling and perspective on the signal of ursodeoxycholic acid, the most hydrophilic bile acid, in the heart. *Biomolecules* 8:159. 10.3390/biom8040159 30486474 PMC6316857

[B10] Hu J. Wang C. Huang X. Yi S. Pan S. Zhang Y. (2021). Gut microbiota-mediated secondary bile acids regulate dendritic cells to attenuate autoimmune uveitis through TGR5 signaling. *Cell Rep.* 36:109726. 10.1016/j.celrep.2021.109726 34551302

[B11] Jain H. Marsool M. Goyal A. Sulaiman S. A. Fatima L. Idrees M. (2024). Unveiling the relationship between gut microbiota and heart failure: Recent understandings and insights. *Curr. Probl. Cardiol.* 49:102179. 10.1016/j.cpcardiol.2023.102179 37923029

[B12] Khan M. S. Shahid I. Bennis A. Rakisheva A. Metra M. Butler J. (2024). Global epidemiology of heart failure. *Nat. Rev. Cardiol.* 21 717–734. 10.1038/s41569-024-01046-6 38926611

[B13] Komorniak N. Pawlus J. Gaweł K. Hawryłkowicz V. Stachowska E. (2024). Cholelithiasis, gut microbiota and bile acids after bariatric surgery-can cholelithiasis be prevented by modulating the microbiota? A literature review. *Nutrients* 16:2551. 10.3390/nu16152551 39125429 PMC11314327

[B14] Li C. Deng L. Pu M. Ye X. Lu Q. (2024). Coptisine alleviates colitis through modulating gut microbiota and inhibiting TXNIP/NLRP3 inflammasome. *J. Ethnopharmacol.* 335:118680. 10.1016/j.jep.2024.118680 39117021

[B15] Li L. Zhong S. J. Hu S. Y. Cheng B. Qiu H. Hu Z. X. (2021). Changes of gut microbiome composition and metabolites associated with hypertensive heart failure rats. *BMC Microbiol.* 21:141. 10.1186/s12866-021-02202-5 33952214 PMC8097775

[B16] Liu B. Ding Z. Xiong J. Heng X. Wang H. Chu W. (2022). Gut microbiota and inflammatory cytokine changes in patients with ankylosing spondylitis. *Biomed. Res. Int.* 2022:1005111. 10.1155/2022/1005111 36033581 PMC9417757

[B17] Liu X. Fassett J. Wei Y. Chen Y. (2013). Regulation of DDAH1 as a potential therapeutic target for treating cardiovascular diseases. *Evid. Based Compl. Alternat. Med.* 2013:619207. 10.1155/2013/619207 23878601 PMC3710625

[B18] Luqman A. Hassan A. Ullah M. Naseem S. Ullah M. Zhang L. (2024). Role of the intestinal microbiome and its therapeutic intervention in cardiovascular disorder. *Front. Immunol.* 15:1321395. 10.3389/fimmu.2024.1321395 38343539 PMC10853344

[B19] Madan S. Mehra M. R. (2020). Gut dysbiosis and heart failure: Navigating the universe within. *Eur. J. Heart Fail.* 22 629–637. 10.1002/ejhf.1792 32168550

[B20] Mayerhofer C. Ueland T. Broch K. Vincent R. P. Cross G. F. Dahl C. P. (2017). Increased secondary/primary bile acid ratio in chronic heart failure. *J. Card. Fail.* 23 666–671. 10.1016/j.cardfail.2017.06.007 28688889

[B21] Nagatomo Y. Tang W. H. (2015). Intersections between microbiome and heart failure: Revisiting the gut hypothesis. *J. Card. Fail.* 21 973–980. 10.1016/j.cardfail.2015.09.017 26435097 PMC4666782

[B22] Nie Y. F. Hu J. Yan X. H. (2015). Cross-talk between bile acids and intestinal microbiota in host metabolism and health. *J. Zhejiang Univ. Sci. B* 16 436–446. 10.1631/jzus.B1400327 26055905 PMC4471595

[B23] Oliphant K. Ali M. D’Souza M. Hughes P. D. Sulakhe D. Wang A. Z. (2021). *Bacteroidota* and *Lachnospiraceae* integration into the gut microbiome at key time points in early life are linked to infant neurodevelopment. *Gut Microbes* 13:1997560. 10.1080/19490976.2021.1997560 34839801 PMC8632288

[B24] Pasini E. Aquilani R. Testa C. Baiardi P. Angioletti S. Boschi F. (2016). Pathogenic gut flora in patients with chronic heart failure. *JACC Heart Fail* 4 220–227. 10.1016/j.jchf.2015.10.009 26682791

[B25] Perino A. Demagny H. Velazquez-Villegas L. Schoonjans K. (2021). Molecular physiology of bile acid signaling in health, disease, and aging. *Physiol. Rev.* 101 683–731. 10.1152/physrev.00049.2019 32790577

[B26] Samsamshariat S. A. Samsamshariat Z. A. Movahed M. R. (2005). A novel method for safe and accurate left anterior descending coronary artery ligation for research in rats. *Cardiovasc. Revasc. Med.* 6 121–123. 10.1016/j.carrev.2005.07.001 16275608

[B27] Sangeethadevi G. Sathibabu Uddandrao V. V. Jansy Isabella R. Saravanan G. Ponmurugan P. Chandrasekaran P. (2022). Attenuation of lipid metabolic abnormalities, proinflammatory cytokines, and matrix metalloproteinase expression by biochanin-A in isoproterenol-induced myocardial infarction in rats. *Drug. Chem. Toxicol.* 45 1951–1962. 10.1080/01480545.2021.1894707 33719799

[B28] Savarese G. Becher P. M. Lund L. H. Seferovic P. Rosano G. Coats A. (2023). Global burden of heart failure: A comprehensive and updated review of epidemiology. *Cardiovasc. Res.* 118 3272–3287. 10.1093/cvr/cvac013 35150240

[B29] Tang W. Li D. Y. Hazen S. L. (2019). Dietary metabolism, the gut microbiome, and heart failure. *Nat. Rev. Cardiol.* 16 137–154. 10.1038/s41569-018-0108-7 30410105 PMC6377322

[B30] Wahlström A. Sayin S. I. Marschall H. U. Bäckhed F. (2016). Intestinal crosstalk between bile acids and microbiota and its impact on host metabolism. *Cell Metab.* 24 41–50. 10.1016/j.cmet.2016.05.005 27320064

[B31] Wang M. N. Cao Y. G. Wei Y. X. Ren Y. J. Liu Y. L. Chen X. (2022). Saffloflavone, a new flavonoid from the flowers of *Carthamus tinctorius* L. and its cardioprotective activity. *Nat. Prod. Res.* 36 3317–3323. 10.1080/14786419.2020.1855167 33432825

[B32] Wang X. Li W. Zhang Y. Sun Q. Cao J. Tan N. (2022). Calycosin as a novel PI3K activator reduces inflammation and fibrosis in heart failure through AKT-IKK/STAT3 axis. *Front. Pharmacol.* 13:828061. 10.3389/fphar.2022.828061 35264961 PMC8899514

[B33] Wang Y. Wang Q. Li C. Lu L. Zhang Q. Zhu R. (2017). A review of chinese herbal medicine for the treatment of chronic heart failure. *Curr. Pharm. Des.* 23 5115–5124. 10.2174/1381612823666170925163427 28950815 PMC6340156

[B34] Wang Z. Liu C. Wei J. Yuan H. Shi M. Zhang F. (2024). Network and experimental pharmacology on mechanism of yixintai regulates the TMAO/PKC/NF-κB signaling pathway in treating heart failure. *Drug Design Dev. Therapy* 18 1415–1438. 10.2147/DDDT.S448140 38707614 PMC11069381

[B35] Winston J. A. Theriot C. M. (2020). Diversification of host bile acids by members of the gut microbiota. *Gut Microbes* 11 158–171. 10.1080/19490976.2019.1674124 31595814 PMC7053883

[B36] Wohlfahrt P. Jenča D. Stehlik J. Melenovský V. Mrázková J. Staněk V. (2023). Heart failure-related quality-of-life impairment after myocardial infarction. *Clin. Res, Cardiol.* 112 39–48. 10.1007/s00392-022-02008-z 35304902

[B37] Yang C. Li X. Hu M. Li T. Jiang L. Zhang Y. (2024). Gut microbiota as predictive biomarker for chronic heart failure in patients with different nutritional risk. *J. Cardiovasc. Transl. Res.* 17 1240–1257. 10.1007/s12265-024-10529-3 38913293

[B38] Yu W. Jiang Y. Xu H. Zhou Y. (2023). The interaction of gut microbiota and heart failure with preserved ejection fraction: From mechanism to potential therapies. *Biomedicines* 11:442. 10.3390/biomedicines11020442 36830978 PMC9953339

[B39] Yuzefpolskaya M. Bohn B. Nasiri M. Zuver A. M. Onat D. D. Royzman E. A. (2020). Gut microbiota, endotoxemia, inflammation, and oxidative stress in patients with heart failure, left ventricular assist device, and transplant. *J. Heart Lung Transplant* 39 880–890. 10.1016/j.healun.2020.02.004 32139154 PMC7423693

[B40] Zhang Q. L. Chen X. H. Zhou S. J. Lei Y. Q. Huang J. S. Chen Q. (2023). Relationship between disorders of the intestinal microbiota and heart failure in infants with congenital heart disease. *Front. Cell Infect. Microbiol.* 13:1152349. 10.3389/fcimb.2023.1152349 36968106 PMC10036851

[B41] Zhang S. Zhou J. Wu W. Zhu Y. Liu X. (2023). The role of bile acids in cardiovascular diseases: From mechanisms to clinical implications. *Aging Dis.* 14 261–282. 10.14336/AD.2022.0817 37008052 PMC10017164

[B42] Zhang Y. Chen L. Hu M. Kim J. J. Lin R. Xu J. (2020). Dietary type 2 resistant starch improves systemic inflammation and intestinal permeability by modulating microbiota and metabolites in aged mice on high-fat diet. *Aging* 12 9173–9187. 10.18632/aging.103187 32452830 PMC7288951

[B43] Zuo L. Chuang C. C. Hemmelgarn B. T. Best T. M. (2015). Heart failure with preserved ejection fraction: Defining the function of ROS and NO. *J. Appl. Physiol.* 119 944–951. 10.1152/japplphysiol.01149.2014 25977452

